# When the crowd gets it wrong – the limits of collective wisdom in machine learning

**DOI:** 10.1038/s41598-025-08273-y

**Published:** 2025-07-01

**Authors:** Kamil P. Orzechowski, Julian Sienkiewicz, Agata Fronczak, Piotr Fronczak

**Affiliations:** Warsaw University of Technology, Faculty of Physics, ul. Koszykowa 75, 00-662 Warsaw, Poland

**Keywords:** Mathematics and computing, Computational science

## Abstract

This study examines collective decision-making dynamics using a machine learning framework, drawing parallels between a previously established synthetic population model and a newly introduced ensemble machine learning counterpart. Grounded in the “wisdom of crowds” principle, the research explores scenarios where the accuracy of group decisions may unexpectedly decrease as group size increases, particularly when individuals share highly correlated information. By replicating these conditions with machine learning ensembles, such as decision trees and support vector machines, the study identifies circumstances where collective accuracy declines, challenging the assumption that larger groups inherently make better decisions. The findings reveal the limitations of collective models in machine learning and provide valuable insights for data-scarce environments.

## Introduction

One of the most recognizable issues in the area of collective decision making is so-called the wisdom of the crowds^1–3^, which highlights the collective judgment of a group as potentially superior to the decisions of any single individual. A significant contribution to a broader understanding of this phenomenon and the general theory of social choice has been made by Nicolas de Condorcet, who in 1785 formulated an argument today well known as de Condorcet’s Jury Theorem^4^. The theorem considers a binary decision-making process in which one of the options is objectively correct, and a group of *N* individuals collectively determines the outcome. Each individual has a probability $p_i$, representing their competence in making the correct decision. The theorem operates under the following assumptions:

- the competences of the individuals are similar (i.e. $q_1 =... = q_N \equiv q$).
- they are statistically independent in their decision-making.
- decisions are determined by a simple majority of votes.

When all of the conditions above are met, the probability of making a correct decision (D) by a group of individuals (judges) is given by:

1 $$\begin{aligned} p^{*}(D) = \sum _{k> \frac{N}{2}}^{N} \genfrac(){0.0pt}1{N}{k} q^{k}(1-q)^{N-k}. \end{aligned}$$

Moreover, when the judges’ competence exceeds the minimum level ($q_i> 0.5$), the probability approaches certainty as the group size increases:

2 $$\begin{aligned} \lim _{N \rightarrow \infty } p^{*}(D) = 1. \end{aligned}$$

This result indicates that, under specific conditions, larger groups are more likely to reach the correct decision. Interestingly, even a group composed of only moderately competent individuals can outperform its most skilled members in terms of accuracy^5,6^.

However, real-world scenarios often deviate from these ideal conditions. For example, individual decisions are frequently influenced by external factors, such as environmental signals, information diffusion, or shared stimuli^7^. For instance, a group of people - voters - usually first get acquainted with relevant information before deciding who among candidates to vote for. This data may refer to a candidate or other topics influencing voters’ choices. For example, a candidate’s participation in a charity event may encourage voters to support a certain candidacy. Conversely, a signal indicating a candidate’s connections to corruption may discourage potential voters from further supporting a particular candidacy.

Individuals obtain vast amounts of information from specific and often common sources. Additionally, the number of these sources can be enormous (possibly even creating an information overload^8,9^) and have a parallel impact on the individuals’ decision: in the real natural environment - for various groups of animals and other organisms^10,11^, or in the media space - for groups and clusters of people^12–15^. The consequence of absorbing information this way may be a highly correlated judgment of individuals - drawing from similar or the same sources.

**Fig. 1:**
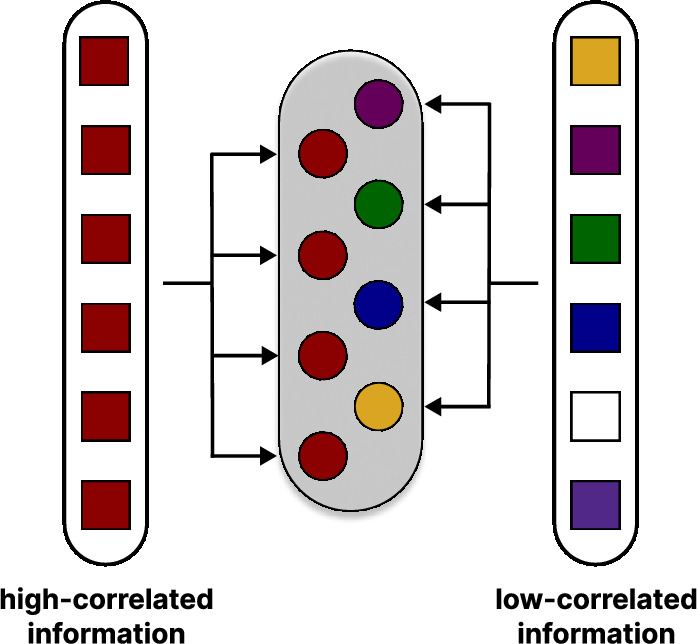
Synthetic model of population in an external environment. The group is exposed to two types of cues. Some individuals (red circles) choose to follow the signal with a high observational correlation, leading to their similar perception. On the other hand, others are more likely to reason differently and have diverse perspectives. Consequently, for each of the *M* decision problems, the group of *N* low- and high-correlated individuals is composed, which makes choice by the majority vote.

Given the above considerations, it is worth considering the impact of correlated information on a group’s collective efficiency. Correlation can undermine the statistical independence of individuals and could have a strong impact on the wisdom of crowds^16,17^. In^7^, this issue is explored using a synthetic model that simulates the collective decision-making process of a group of individuals in a complex environment. According to the described strategy, each individual in the group uses information with varying degrees of observed correlation in the environment. The unit decides on a given issue according to a specific procedure. All decisions are then aggregated into a majority decision, constituting the entire group’s verdict (see Fig. 1). The study demonstrates that external stimulation could significantly impact the efficiency of the group decision-making process, revealing scenarios where collective accuracy may decrease as group size increases. This finding challenges the assumption that the wisdom of the crowds is always an optimal solution.

Being a statistical process, the wisdom of crowds has inspired numerous applications in modern research^18^, many of which are rooted in ideas originally proposed by de Condorcet. A relevant example is the separate area of research on artificial intelligence methods. The assumption of the existence of “weak” individuals (i.e., models - quantitative statistical methods) who, in a group, are able to make collective decisions more effectively constitutes the backbone of the field of so-called ensemble machine learning^19^. Considering that there are systems in which the collective efficiency of a group, under certain conditions, decreases with its size, in the context of the issue under consideration, a natural question arises: is it possible to reproduce such results by ensembles of machine learning models? Although the system described above is an implementation of a synthetic model of the behavior of a population of individuals, the results suggest the weakness of the wisdom of crowds in certain situations.

These findings inspire us to delve deeper into this topic as taking into consideration ensembles of machine learning models in collective decision making with correlated information might be an interesting research subject. Our motivation here is to identify analogous patterns between artificial decision-making systems (those with a certain accuracy that can be determined quantitatively, such as the synthetic model introduced above) and statistical predictive models - machine learning algorithms - trying to uncover similar features. Although creating ensembles makes sense for most of the known prediction models^20^ and our research included ensembles of different well-known machine learning algorithms, we decided to base our eventual outcomes on i. decision trees^21^, valued for their interpretability, and ii. support vector machines^22,23^, known for their high performance and reduced risk of overfitting. By investigating these ensembles, we aim to deepen our understanding of collective decision-making in artificial systems and identify parallels with natural group behavior.

## Methods

### Synthetic model of a population in a complex environment

The wisdom of crowds emerges when low-correlated individuals, possessing minimal competence, form the majority of a population. In such cases, their collective decision-making becomes increasingly accurate as the group size grows^7,24^. However, there are scenarios where the underlying assumptions of this phenomenon are not fully met or are only partially satisfied. An example of research addressing such scenarios is a synthetic model of population behavior initially proposed by Kao and Couzin in^7^, which demonstrates that a group’s collective accuracy does not always improve with size under specific conditions.

To better understand this model, let us revisit its assumptions and key results. The model considers a group of individuals operating in an environment where two parallel and distinct environmental signals, with varying degrees of correlation and reliability, are present (see Fig. 1). The population, consisting of *N* members, faces a binary decision problem. The judgment is made initially by each individual and then aggregated to a collective decision using a simple majority rule. The model algorithm can be described as follows:

A set of *M* decisions to be made is considered, for which the set of solutions is two-element, and one of the options is known to be correct. For each of the issues considered, the population is unique and formed as follows:

- individuals in the population with probability *p* are independent units. They all make the correct choice regarding a given decision problem by drawing each time with a predefined probability $r_L$, which is a fixed parameter of the model. This means that all independent units are completely uncorrelated (i.e., the following scenario is considered: the observation for each individual is independent and identically distributed).
- on the other hand, all dependent units make a correct decision according to the probability $r_H$, which is a fixed parameter of the model. Unlike the independent units, here the drawing is performed only once in advance for each issue, indicating the corresponding decision. Consequently, all dependent units are perfectly correlated (i.e., the following scenario is considered: the observation for each individual is exactly the same).
- when all individuals in the group have already expressed their opinion, their votes are collectively aggregated, and the group, as a unified entity, makes the final decision by majority vote.

The model presented above is an implementation of the system that lies upon the three key parameters:

- $p \in \langle 0,1 \rangle$ - the probability that individuals rely on low-correlation cues (voting strategy) when confronted with an external environment. Independent individuals relying on low-correlation signals base their judgments on autonomous and diverse sources of information. On the other hand, dependent individuals relying on high-correlation signals base their decisions on information widely shared among group members, reducing diversity.
- $r_L \in \langle 0.5,1 \rangle$ - the probability of making a correct decision by an independent individual, equivalent to the reliability of an environmental signal with low correlation.
- $r_H \in \langle 0.5,1 \rangle$ - the probability of making a correct decision by a dependent individual, equivalent to the reliability of an environmental signal with high correlation.

**Fig. 2:**
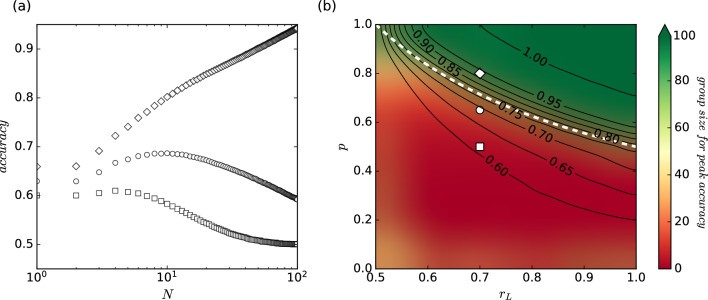
(**a**) Variability in collective accuracy (group’s effectiveness by a majority vote, see Eq. 4) depending on group size (N) and the wisdom of crowds’ presence. The outcomes correspond in the shape of the markers to the three white points in the right panel, indicating the pairs of parameters ($r_{L}$,*p*). The white and green points demonstrate the cases where the collective accuracy of certain group sizes surpasses that of infinitely large groups. (**b**) Environmental and behavioral space of parameters for the synthetic model. The colors on the map indicate the group size for which maximum accuracy could be achieved. The area colored in green denotes a regime of parameters for which the wisdom of crowds can be observed. This is where the maximum accuracy would be obtained for the largest group size. The red-colored region is related to the regime where this observation does not occur i.e., the smaller group has the highest accuracy, which then starts to decrease with the higher number of individuals. The white dotted line symbolizes the critical boundary between the regimes (see Eq. 3). The contours on the map show the maximum accuracy that can be achieved for the selected set of parameters. Results were obtained for $r_H = 0.5$ and averaged over 100000 decision problems. The figures are outcomes of simulation of the model established by Kao and Couzin, and prepared by the authors of this manuscript. Legend: *p* - the probability that individuals rely on low-correlation cues (voting strategy) when confronted with an external environment, $r_L$ - the probability of making a correct decision by an independent individual, $r_H$ - the probability of making a correct decision by a dependent individual.

Following the aforementioned features, there are two areas, which might be defined in the parameter space (see Fig. 2): i. the first one, where the wisdom of crowds occurs, and ii. the second one in which this phenomenon is impossible. The critical boundary is based on the following condition: un-/low-correlated individuals must constitute the majority of the decision-making group. It means that the wisdom of the crowds can be observed only for *p* as defined (following Kao and Couzin in^7^) below:

3 $$\begin{aligned} p r_{L} N> \frac{1}{2} N \Rightarrow p> \frac{1}{2 r_{L}} \end{aligned}$$

The white dotted line in Fig. 2(b) symbolizes the critical boundary between the regimes of parameter space $(r_L,p)$.

The model provides output in the form of collective decisions regarding predicted labels that could be juxtaposed with actual classes in the test set, which were drawn from a uniform two-point discrete distribution. Further evaluation was performed using the most well-known metric - accuracy, which generally indicates how often a model is correct. It is defined as shown below:

4 $$\begin{aligned} accuracy = \frac{TP + TN}{TP + TN + FP + FN}, \end{aligned}$$

where TP and TN are defined as a number of cases related to the correct prediction of positive and negative classes, respectively. On the other hand, FP and FN indicate a number of cases connected with the wrong prediction of positive and negative classes, respectively.

It should be noted that the contour lines in Fig. 2b (and later in Figures 5b and 5d) are only approximate, particularly in the region above the critical line (i.e., in the *wisdom of the crowd* region). According to Eq. (2), accuracy should be equal to one across this entire parameter range. However, as seen slightly above the critical line, the observed accuracy values are lower. This discrepancy arises because, for small values of $r_L$, or when the proportion of dependent judges is high, achieving maximum accuracy would require a significantly larger number of judges than the predefined in our simulations $N_{max} = 100$. This is due to the fact that the convergence of the limit in Eq. (2) in these cases is very slow.

### Ensemble model via machine learning approach

As mentioned in the introduction, a natural question arises: can synthetic population models associated with collective decision-making processes be replicated using more complex systems? The proposed approach, inspired by the methods discussed in the previous section, offers a slightly different perspective on this topic–examining the collective performance of a group. The leitmotif of our solution is based on the original assumption of de Condorcet, who formulated his famous theorem concerning a group of judges making a decision during a court hearing. Judges may have varied experience and knowledge. What is important is that their number is limited, as indicated by the predefined number of seats at the hearing.

In our approach, we conceptualize an ensemble of judges in such an environment. However, these judges are not individuals but machine learning models^25–28^ (henceforth, the terms *models* and *judges* will be used interchangeably). Here, we also refer to a binary decision problem - in this case, binary classification in supervised learning^29,30^. The algorithm for this model is as follows:

1. a classification dataset is considered, divided into training and test subsets^31^ in a ratio of 3:7. It should be highlighted that the division between training and test sets is of lower significance in the judges’ training context. This is because the factual efficiency of independent and dependent judges depends on the number of training samples $n_L$ and $n_H$, respectively (see Table 1). We present results for several different train-test splits as part of the supplementary material, showing that the impact of a certain split on the eventual results is of minor significance.
2. initially, two groups of available judges are created, each large enough to allow repeated sampling in subsequent steps (see Step 4). The difference between groups is reflected in the expected correlation among their members. The first group consists of *L* models trained independently using distinct training data. The second group consists of *H* models trained on the same data, representing a shared source of knowledge or information (Please note that classification models, even when trained on the same dataset, can vary due to inherent stochastic elements in their training process. For instance, decision trees are constructed by selecting splits based on criteria such as Gini impurity or information gain. When multiple splits yield similar scores, the algorithm may select one arbitrarily, resulting in variations in the tree structure.). Consequently (and by analogy to the synthetic model), we will refer to the former group as independent judges and the latter as dependent judges. The specific sizes of groups *L* and *H* are not critical. They simply need to be large enough to allow, in extreme cases, the random selection of *N* distinct individuals from each group ($L\ge N$, $H\ge N$). Model training is carried out using the classic bootstrap method^32,33^, where the drawing concerns only observations while maintaining an identical set of features, and the classes of training samples are distributed evenly. It should be emphasized that the parameters of the synthetic model (i.e., the reliability of low/highly correlated signals, $r_L$, and $r_H$, respectively) have their mapped equivalents in the form of the number of training samples ($n_{L}$ and $n_{H}$). Consequently, the probability of making a correct decision by independent and dependent judges is more likely to increase to a certain extent with the number of samples on which they are trained^34,35^. Both ensembles are constant and do not change over the further steps. We are aware that judges in our predictive model are trained on a small volume of samples; however, this is done intentionally. The comfort of having access to the amount of data, which is seen as high enough, is not always on the table. We try to show this using a specific ensemble model in line with the leitmotif of our work – the limits of collective wisdom in machine learning.
3. each sample in the test set (of size *M*) represents an independent court hearing (decision-making process), during which *N* seats are occupied by judges drawn from both groups.
4. for each hearing:
  with probability *p* seats are occupied by models drawn from the group of *L* independent judges. Otherwise, they are filled by the dependent ones.
  each of the *N* judges makes a decision (prediction) based on the information from the hearing (test sample with a certain number of features).
  when all the judges have already expressed their opinions, the votes are aggregated, and the final decision by majority vote is made.

**Fig. 3:**
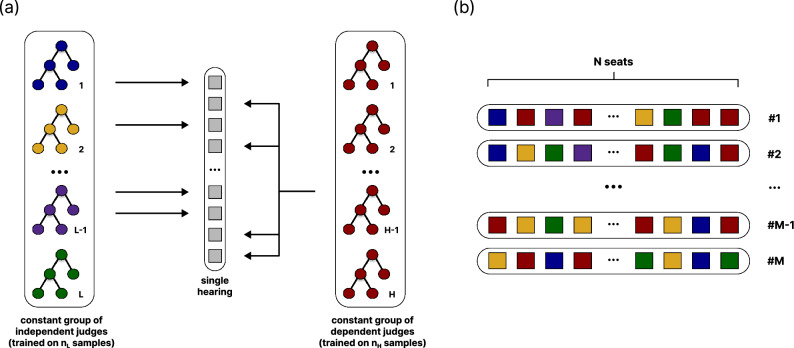
A predictive model based on the ensembles of machine learning algorithms. (**a**) For each test sample, the group of *N* models is formed, which serves as an equivalent of the court hearing scenario. They are sampled from two separate groups of independent and dependent judges of size *L* and *H*, respectively. In terms of the ensemble, two training approaches are utilized. Some models (red ones) are trained on the same training data, while others use diverse data. Eventually, all of them make predictions and provide the majority decision. (**b**) The process is repeated for each sample within the test set of size *M*. All parameters of the predictive model alongside descriptions are available in the Table 1.

The schematic of the entire algorithm is presented in Fig. 3. For clarity, all parameters of the model were gathered and described in the Table 1. Furthermore, both algorithms - the original one by Kao and Couzin and the one adopted by us for classification problems - are presented in a way that highlights their similarities and differences as part of the supplementary material.

**Table 1:** Parameters of the predictive model alongside descriptions.

**Parameter**	**Meaning**
*L*	size of the group of available independent judges
*H*	size of the group of available dependent judges
*p*	probability that a seat during the hearing is occupied by a judge from the group of independent judges
$n_{L}$	# of training samples for independent judges
$n_{H}$	# of training samples for dependent judges
*N*	# of seats per hearing (judges per decision/group size)
*M*	# of hearings (size of the test set)

Here, we decided to present the results utilizing the accuracy metric (excluding metrics such as precision and recall) because accuracy was specifically used in the original research on the synthetic model^7^, and our aim was to highlight further similarities between the models while maintaining a consistent and reliable reference point.

## Datasets

In this research, a binary decision-making problem was analyzed, leading us to work with datasets specifically designed for binary classification in supervised learning. Ultimately, we selected two datasets to determine whether the results of the synthetic model could be reproduced using machine learning models.

**Fig. 4:**
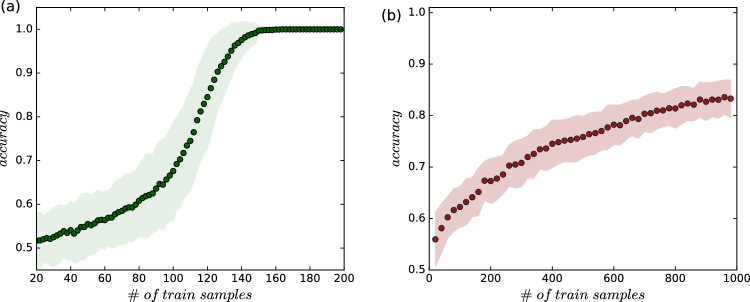
The optimal learning curves (test sets) obtained for the chosen datasets and corresponding models. (**a**) Support Vector Classifier (SVC) with Radial Basis Function (RBF) kernel used on Circles Clusters dataset. (**b**) Decision Tree Classifier (DTC) used on Secondary Mushroom dataset. Results obtained for CV = 100. The bars in both panels represent the standard deviations.

While over a dozen classification datasets (both natural and synthetic) were evaluated during the study, the final selection was made based on the unique requirements of our approach and the phenomena under investigation. Our goal was to ensure that the models in the ensembles (as described in the previous section) would not achieve excessively high accuracy with a small number of training samples. Such performance would imply that the models were too effective, preventing the observation of the effects we aimed to study. Two datasets (see Table 2) exhibit slower learning curve dynamics (see Fig. 4) and allow for relatively simple manipulation of the number of samples–flexibility that was not afforded by other datasets considered. Additionally, the difference between the maximum accuracy achieved at intermediate judge group sizes and the minimum accuracy (observed at minimum $N = 1$ or maximum $N = 100$) is significant for those two datasets. This feature distinguishes them from other datasets for which the narrow scope of accuracy variation has been observed. We present detailed information about all datasets and how they were used, along with the selected corresponding heatmaps as part of the supplementary material.

**Table 2:** Classification datasets used during the eventual research phase. The Mushroom dataset was appropriately pre-processed, considering the standard pipeline related to quantitative variables for binary classification tasks. Clusters dataset was processed for factor = 0.85.

**Dataset**	**# of features**	**# of samples**	**class 0**	**class 1**	**M**
Circles Clusters	2	5000	2500	2500	3500
Secondary Mushroom	9	54035	24360	29675	37825

- Circles Clusters (source: https://scikit-learn.org/1.5/modules/generated/sklearn.datasets.make_circles.html) make_circles - an artificial dataset utilising for clustering and classification tasks in 2D.
- Secondary Mushroom (source: https://www.kaggle.com/datasets/prishasawhney/mushroom-dataset) - prediction of whether simulated mushrooms are edible and poisonous.

## Results

### Collective decision-making as part of machine learning approach

**Fig. 5:**
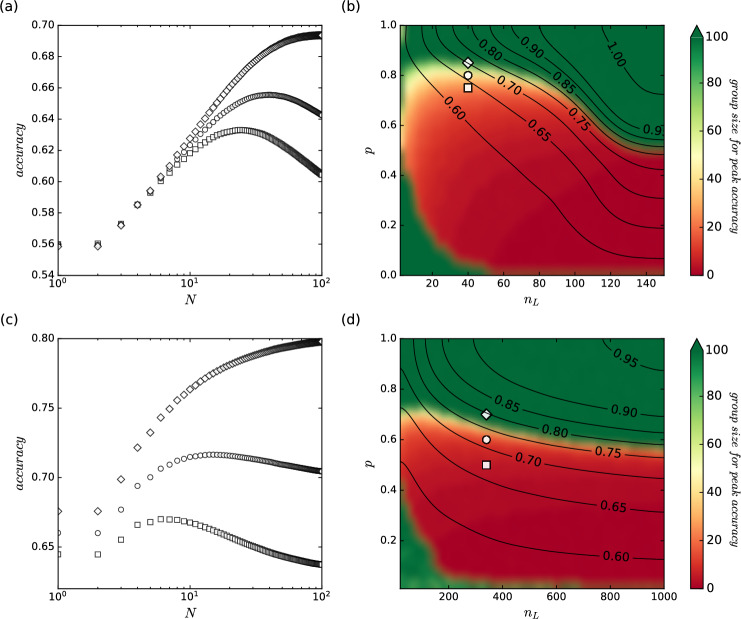
Collective accuracy varies depending on group size (N) and the wisdom of crowds’ presence. (**a**) Results for SVC based on Circles Clusters dataset ($n_H = 50$, $L = 1000$, $H = 100$, outcomes averaged over 1000 runs of simulation). (**b**) Two regimes in environmental and behavioral space for the ensembles consisted of SVC judges on the Circles Clusters dataset. (**c**) Results for the DTC based on the Secondary Mushroom dataset ($n_H = 30$, $L = 1000$, $H = 100$, outcomes averaged over 100 runs of simulation). (**d**) Two regimes in environmental and behavioral space for the ensembles consisted of DTC judges on the Secondary Mushroom dataset. The outcomes in panels (**a**, **c**) match in the shape of the markers to the three white points in the right panels, indicating the pairs of parameters ($n_{L}$, *p*). The colors within panels (**b**, **d**) indicate the group size for which maximum accuracy could be achieved. The areas colored in green denote regimes of parameters for which the wisdom of crowds can be observed. This is where the maximum accuracy would be obtained for the largest group size. The red-colored regions are related to the regimes where this observation does not occur i.e., the smaller group has the highest accuracy, which then starts to decrease with the higher number of individuals. The contours show the maximum accuracy that can be achieved for the selected set of parameters. All parameters of the predictive model alongside descriptions are available in the Table 1.

This subsection summarizes the results obtained in our analysis. The phenomenon of collective decision-making from the perspective of the machine learning approach is shown in Fig. 5. This figure should be analyzed in relation to the analogous Fig. 2, which pertains to the synthetic model. Panels (a) and (c) depict how collective accuracy evolves with group size. Notably, the results indicate (see, e.g., white and green points in these panels) that not all individuals in a group are necessary to achieve the best decision. In fact, a specific, optimal group size exists that guarantees the highest accuracy.

However, this is just a fragment of the broader landscape regarding the potential weaknesses of the wisdom of crowds under specific conditions. A more thorough understanding requires examining how collective accuracy behaves across the entire parameter space. Panels (b) and (d) of Fig. 5 present the results for the machine learning-based predictive model, mapping the global behavior of collective accuracy. It is worth recalling that, unlike the synthetic model, which directly uses $r_L$ and $r_H$ parameters, the predictive model employs their mapped equivalents ($n_L$ and $n_H$), represented as the sizes of the training samples used to train the models/judges in the group. These figures showcase the global behavior of the two predictive models (SVC and DTC) as a function of the training sample size for dependent judges.

One noticeable difference between the phase diagram of the synthetic model (Fig. 2(b)) and that of the predictive model (Figs. 5(b) and 5(d)) is the lower-left region, where the wisdom of crowds phenomenon occurs in the predictive model. However, considering the very low maximum achievable accuracy in this area (indicated by contour lines on the panels), it can be assumed that this is merely an artifact resulting from the fact that accuracy increases slightly–from an initial value of 50% to just over 50%–as the number of judges grows. Therefore, this region is insignificant from the perspective of applying classification models.

The results highlight the existence of a framework indicating parameter regimes where smaller groups outperform larger ones in decision-making. This phenomenon may arise because a high correlation among dependent judges introduces redundancy in information and shared errors. The models contribute similar perspectives and replicate mistakes stemming from training on the same data, significantly impacting the group’s overall performance. Conversely, with low correlation, challenges may emerge in integrating diverse opinions. While diversity can enhance decision-making, excessively large groups can lead to inefficiencies and disorder, especially given the limitations in predictive model accuracy.

Following the synthetic model’s outcomes (see Fig. 2), our system also indicates the presence of a boundary between areas within which the wisdom of the crowds occurs or disappears in parameter space (see Fig. 5). On this basis, it can be concluded that the predictive model replicates some properties of the synthetic model. However, its decision boundary is much more sensitive to parameter changes.

### Failure of the wisdom of crowds in machine learning approach

This subsection illustrates insights on what can have a potential impact on the failure of the wisdom of the crowds in the machine learning and ensembling space. It is worth noting that the ensemble of machine learning models is a much more complex system than the one represented by the synthetic model. Therefore, there are areas where a compromise had to be sought between the specificity of our approach and the conditions present in the original studies.

This compromise primarily pertains to the correlation between individuals, referred to in our approach as “dependent judges”. These judges represent highly correlated individuals drawing analogous information from the same source. Such individuals consequently make almost identical decisions (minor differences between them arise from the inherent stochastic elements involved in constructing the classification model.). With this in mind, we made an effort to examine the impact of model correlation in our approach on the response of the entire decision system. This was done by increasing the variance in the training data of models/dependent judges in the following way: The dataset of size $n_H$ used to train each classification model corresponding to a dependent judge consisted of two parts: one shared among all such judges ($\alpha n_H$) and another portion of training data selected individually for each judge ($(1-\alpha )n_H$). The larger this individual portion, the weaker the correlations between the judges in this group.

**Fig. 6:**
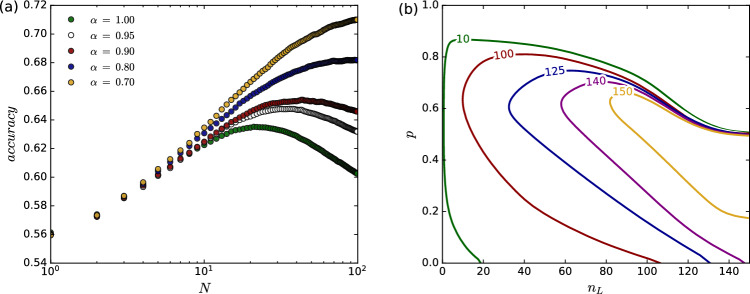
(**a**) Collective accuracy depending on the degree of correlation (emphasized in different colors) between models within the group of dependent judges. Results obtained for the configuration of parameters ($p = 0.75$, $n_L = 40$, $n_H = 50$) represented by the green point in the panel (**b**) of Fig. 5(**b**) Variation of the wisdom of crowds breaking region (boundaries represented in the form of contours) depending on the number of training samples $n_H \in \{10, 100, 125, 140, 150\}$ for the group of dependent judges. All outcomes were averaged over 100 runs of simulation. All parameters of the predictive model alongside descriptions are available in the Table 1.

The results we obtained (see Fig. 6(a)) indicate that reducing correlation among dependent judges strongly affects the behavior of the entire system. As the degree of cross-correlation in the decision-making process decreases, the effects described in this work–such as optimal group size–diminish, giving way to the classical wisdom of the crowd phenomenon.

Moreover, Kao and Couzin^7^ demonstrated that in their synthetic model, the higher the probability of highly correlated individuals making a correct decision, the noisier the actual decision boundary becomes, particularly in the range where $r_L < r_H$. As a result, the region corresponding to the optimal finite group size, where the wisdom of crowds fails, shrinks. Similar results can also be observed in our approach. Fig. 6(b) illustrates how the number of samples $n_{H}$ used for training dependent judges influences the system’s global behavior. It is evident that as $n_{H}$ increases, the region where the wisdom of the crowds breaks down effectively vanishes. Figure 6(b) also shows how the parameter space reacts to variations in training samples for independent judges ($n_L$). On the other hand, taking into account the systems with limited access to the data, increasing the number of training samples would cause: i. overlap of train sets (higher correlation between models) and ii. their increased competence. As a result, the phenomenon under consideration would disappear.

The negative impact of homogeneity on the performance of collective decision-making processes is a significant area of research. For example, in the study^36^, the importance of diversity in classifier ensembles is emphasized as a critical factor for improving the accuracy of ensemble methods like bagging and boosting. The authors examined various diversity measures, highlighting that bagging struggles to generate sufficient diversity for stable classifiers, such as linear classifiers, leading to worse ensemble performance. A different approach is presented in work^37^, where diversity in neural network ensembles is enhanced using negative correlation, which forces models to specialize in different aspects of the problem. In this approach, an additional term is added to the cost function of each model in the ensemble, penalizing the positive correlation between their outputs. The models aim to minimize their own prediction error while simultaneously being as uncorrelated as possible with others. As a result, the group becomes more robust to errors and performs better on new, unseen data. In another study^38^, examined SVM ensembles (e.g., bagging with negative correlation) and found that introducing this concept increased diversity while reducing redundancy among models. The approaches described above align with our observations that excessive correlation among decision-making units can reduce group efficiency. Implementing negative correlation in SVM ensembles, in turn, offers valuable inspiration for further research into optimizing collective decision-making in machine learning systems, such as those analyzed in our study. This having been said, we still need to underline that our study, unlike the above-described works, focuses rather on the importance of correlated inputs (data) and not outputs (votings), although, in fact, these two are very strongly connected in our approach.

## Summary and concluding remarks

Although the phenomenon of the wisdom of crowds has been recognized since the 18th century^4^ and extensively analyzed across various fields of science^16,39,40^, there are still areas where it has not been systematically researched. It is worth noting that the concept presented by de Condorcet itself described an extremely idealized system, showing the lack of statistical correlation between individuals–a condition rarely observed in natural systems such as ecosystems or societies^41^. In machine learning, these motifs are also quite entrenched in the form of ensemble learning techniques^42,43^. However, some nuances regarding simple models and the results they return can lead to non-obvious results.

As demonstrated in this work, it is possible to reproduce specific patterns observed in fully virtual synthetic models that describe decision-making mechanisms. We achieved this using our approach, a predictive model (inspired by its synthetic prototype) based on classic machine learning techniques - support vector machines and decision trees. In order to show a more profound similarity between the synthetic and predictive models, we did not focus only on collective accuracy, which, although the flagship observation among the presented ones, is not the only source of interesting information. The overall culmination of our observations was to examine the results returned in the entire available opinion space. The results of the synthetic model and our approach showed significant similarity and clearly defined critical limits, although these were not identical. It may be due to simple facts. The synthetic model is an idealized implementation of a system in which, when all conditions are met, individuals make specific decisions with 100% certainty. In the case of predictive models, however, this situation is highly different, and machine learning models are at risk of making errors even when all conditions in the system are met. It is influenced by many factors because no predictive model is perfect and can only generalize to a certain extent^44,45^. Moreover, the behavior of such a model is also significantly influenced by the data on which it operates, its quality, completeness, and method of processing^46^.

While the findings presented in this paper are based on a single system where the wisdom of crowds demonstrates specific vulnerabilities, our intent was not to introduce new methodologies. Instead, our primary goal was to show that the phenomenon of declining collective accuracy with increasing group size can also manifest in predictive models under appropriate system conditions. These results suggest that employing more models and/or computational units in a team-based approach does not necessarily yield better outcomes. On the contrary, smaller group sizes with fewer resources may sometimes deliver superior results. This work offers valuable insights and highlights potential implications. Although the described phenomena are not ubiquitous and require the convergence of many cases to occur, statistically, they are plausible. The studies were conducted on over a dozen of binary classification datasets, but replicable results were observed only in datasets that met specific constraints, such as slow dynamics of learning curves and, in most cases, near-perfect correlation among models in the group of dependent judges. This may particularly impact research areas where there are data shortages or almost no data. However, we are aware that this requires further in-depth research. The practical outcome of our study is that ensemble learning approaches should incorporate mechanisms to monitor and mitigate correlation among models. For example, instead of simply increasing ensemble size, models should be chosen to maximize independence, such as training on diverse subsets of data or using algorithms with complementary decision boundaries. Assigning lower influence to models that exhibit strong correlation with others can prevent the dominance of misleading signals. Finally, beyond traditional accuracy metrics, incorporating measures of model agreement and redundancy can help identify conditions under which collective performance may deteriorate. Future research could explore automated techniques for detecting and responding to potential wisdom-of-crowds breakdowns in predictive modeling applications. Another line of investigation would be to tackle the problem of optimal use of a single dataset to find out if training multiple models on different subsamples thereof brings better results than using just one model on the entire sample. All these pertinent research questions are out of the scope of the current study but should be undertaken to further understand the limits of ensemble methods.

## Supplementary Information

Supplementary Information.

## Data Availability

The code that supports the findings of this study is available from the corresponding author upon request.
