# Supplementary material for: When the crowd gets it wrong – the limits of collective wisdom in machine learning
Source: Sci Rep. 2025 Jul 1;15:22139. doi: 10.1038/s41598-025-08273-y (PMC12216932; doi:10.1038/s41598-025-08273-y)
Supplement: Supplementary file 1 — Supplementary Information. [file 41598_2025_8273_MOESM1_ESM.pdf]

Supplementary material for
"When the crowd gets it wrong – The limits of collective wisdom in machine learning"

Kamil P. Orzechowski, Julian Sienkiewicz, Agata Fronczak, and Piotr Fronczak

1. Summary of the analyzed datasets

During the research, a binary decision-making problem was considered. Consequently, the datasets we worked on are dedicated to binary classification in supervised learning. In this work, in order to check whether reproducing the results of the synthetic model is possible in the case of decision trees, we used 22 datasets in total. Each was appropriately pre-processed, considering the transformation of quantitative and qualitative variables. We tried to create a relatively universal pipeline for the purpose of future research.

Dataset	Features	Samples	Used features	Used samples	C0	C1
Bank Marketing	17	11162	17	11162	5873	5289
Cancer Prediction	12	1000	12	1000	776	224
Titanic	14	1309	14	1309	809	500
Stroke Prediction	12	5110	12	5110	4861	249
Diabetes	9	768	9	768	500	268
Travel Insurance	12	62288	12	62288	49830	12458
Adult Census Income	15	32561	15	30162	22654	7508
Covid19	10	5834794	10	40000	20000	20000
Car Evaluation	7	1728	7	1728	1210	518
Employee Turnover	10	14999	10	14999	11428	3571
Hotel Booking	32	119390	30	102894	62733	40161
Airline Passengers	25	129880	23	129487	73225	56262
Paris Housing	18	10000	18	10000	8735	1265
Sexual Abuse Awareness	9	3002	9	3002	1711	1291
Weather Australia	25	25000	23	6291	4796	1495
Gladiator	29	746386	26	60000	30000	30000
Circles Clusters	2	5000	2	5000	2500	2500
Secondary Mushroom	9	54035	9	54035	24360	29675
Spambase	57	4601	57	4601	2788	1813
Banknote Authentication	4	1372	4	1372	762	610
Blood Transfusion Service Center	4	748	4	748	570	178
MAGIC Gamma Telescope	10	19020	10	19020	12332	6688

Table 1: Classification datasets used during the research phase. All sets were adjusted to binary classification tasks. Smaller numbers of used features and samples were used because of i. gaps or inconsistencies in the data, ii. too extensive size of some datasets.

- Bank Marketing (source: www.kaggle.com/datasets/janiobachmann/bank-marketing-dataset/) - prediction of term deposit subscriptions.
- Cancer Prediction (source: www.kaggle.com/datasets/fdcellat/cancer-prediction-dataset/) - lung cancer prediction survey - synthetic collection of responses gathered from a university-conducted survey.
- Titanic (source: www.kaggle.com/datasets/marouandaghmoumi/titanic-dataset/) - information about the passengers aboard the RMS Titanic which sank on its maiden voyage in 1912.
- Stroke Prediction (source: www.kaggle.com/datasets/fedesoriano/stroke-prediction-dataset/) - prediction whether a patient is likely to get stroke based on clinical features.
- Diabetes (source: www.kaggle.com/datasets/mathchi/diabetes-data-set/) - prediction whether a patient has diabetes based on diagnostic measurements.

- Travel Insurance (source: https://github.com/Athpr123/Binary-Classification-Using-Machine-learning/) - prediction whether to sanction the insurance claim or not.
- Adult Census Income (source: https://www.kaggle.com/datasets/uciml/adult-census-income/) - prediction whether a person makes over $50K a year.
- Covid19 (source: https://www.kaggle.com/code/mykeysid10/detection-of-covid19-cases-using-ml/) - first tests of Israel's citizens tested for covid, the test results and the indications for the test.
- Car Evaluation (source: www.kaggle.com/datasets/elikplim/car-evaluation-data-set/) - evaluation of cars' condition. The original dataset contained four ordinal classes: unass, acc, good, v-good defining condition of cars. For our purposes, the first three classes have been merged into one: acc. Consequently, we obtained a binary dataset.
- Employee Turnover (source: https://www.kaggle.com/code/serkanp/employee-turnover-prediction/input/) - prediction whether an employee is going to leave the organization in the upcoming period.
- Hotel Booking (source: www.kaggle.com/datasets/jessemostipak/hotel-booking-demand/) - prediction of cancellation of booking based on various booking information between two hotels: a city hotel and a resort hotel.
- Airline Passengers (source: https://www.kaggle.com/datasets/teejmahal20/airline-passenger-satisfaction/) - results of survey useful in prediction of customers' satisfaction for an Airline.
- Paris Housing (source: https://www.kaggle.com/datasets/mssmartypants/paris-housing-classification/) - prediction whether a house's status is luxury or basic.
- Sexual Abuse Awareness (source: https://www.kaggle.com/datasets/sahasourav17/child-sexual-abuse-awareness-knowledge-level/) - prediction of people's level of knowledge regarding child sexual abuse.
- Weather Australia (source: https://www.kaggle.com/datasets/rever3nd/weather-data/) - prediction whether it will rain or not the next day based on records from different areas of Australia.
- Gladiator (source: https://www.kaggle.com/datasets/anthonytherrien/gladiator-combat-records-and-profiles-dataset/) - prediction whether a gladiator survives a duel or not based on personal information, combat statistics and special skills.
- Circles Clusters (source: https://scikit-learn.org/1.5/modules/generated/sklearn.datasets.make_circles.html) make_circles - an artificial dataset utilising for clustering and classification tasks in 2D.
- Secondary Mushroom (source: https://www.kaggle.com/datasets/prishasawhney/mushroom-dataset) - prediction of whether simulated mushrooms are edible and poisonous.
- Spambase (source: https://archive.ics.uci.edu/dataset/94/spambase) - classifying email as spam or non-spam.
- Banknote Authentication (source: https://archive.ics.uci.edu/dataset/267/banknote+authentication) - data were extracted from images that were taken for the evaluation of an authentication procedure for banknotes.
- Blood Transfusion Service Center (source: https://archive.ics.uci.edu/dataset/176/blood+transfusion+service+center) - prediction whether a donor donated blood in March 2007 based on donor database of Blood Transfusion Service Center in Hsin-Chu City in Taiwan.
- MAGIC Gamma Telescope (source: https://archive.ics.uci.edu/dataset/159/magic+gamma+telescope) - discrimination of photons caused by primary gammas (signal) from the images of hadronic showers initiated by cosmic rays in the upper atmosphere (background).

2. Heatmaps of selected datasets

Fig. S1 presents the results of the approach discussed in this work for SVC models applied to four additional datasets: Airline Passengers, Bank Marketing, Car Evaluation, and Adult Census Income. The figure description is identical to that of Fig. 5 in the main text.

In panel (b), in addition to the standard three points representing selected parameter sets—used for the plots in the left column—an additional point, $(r_L, p) = (10, 0.1)$, has been marked. In this region of the parameter space, as the number of judges increases, accuracy rapidly reaches its maximum value, which is very close to the accuracy of a single dependent judge. As a result, this maximum remains relatively low, and the dependency is very flat. Although the plot obtained for this parameter set aligns with the definition of the wisdom of the crowd, it is not relevant for practical applications.

It is worth noting the narrow range of accuracy variation across the datasets presented here. This pattern holds for most of the datasets we analyzed. However, two datasets stand out: Circles Clusters and Secondary Mushroom, whose results are discussed in the main text. For these two datasets, the difference between the maximum accuracy achieved at intermediate judge group sizes and the minimum accuracy (observed at minimum $N = 1$ or maximum $N = 100$) is significant. This highlights the effect of the most effective prediction occurring at a moderate number of judges.

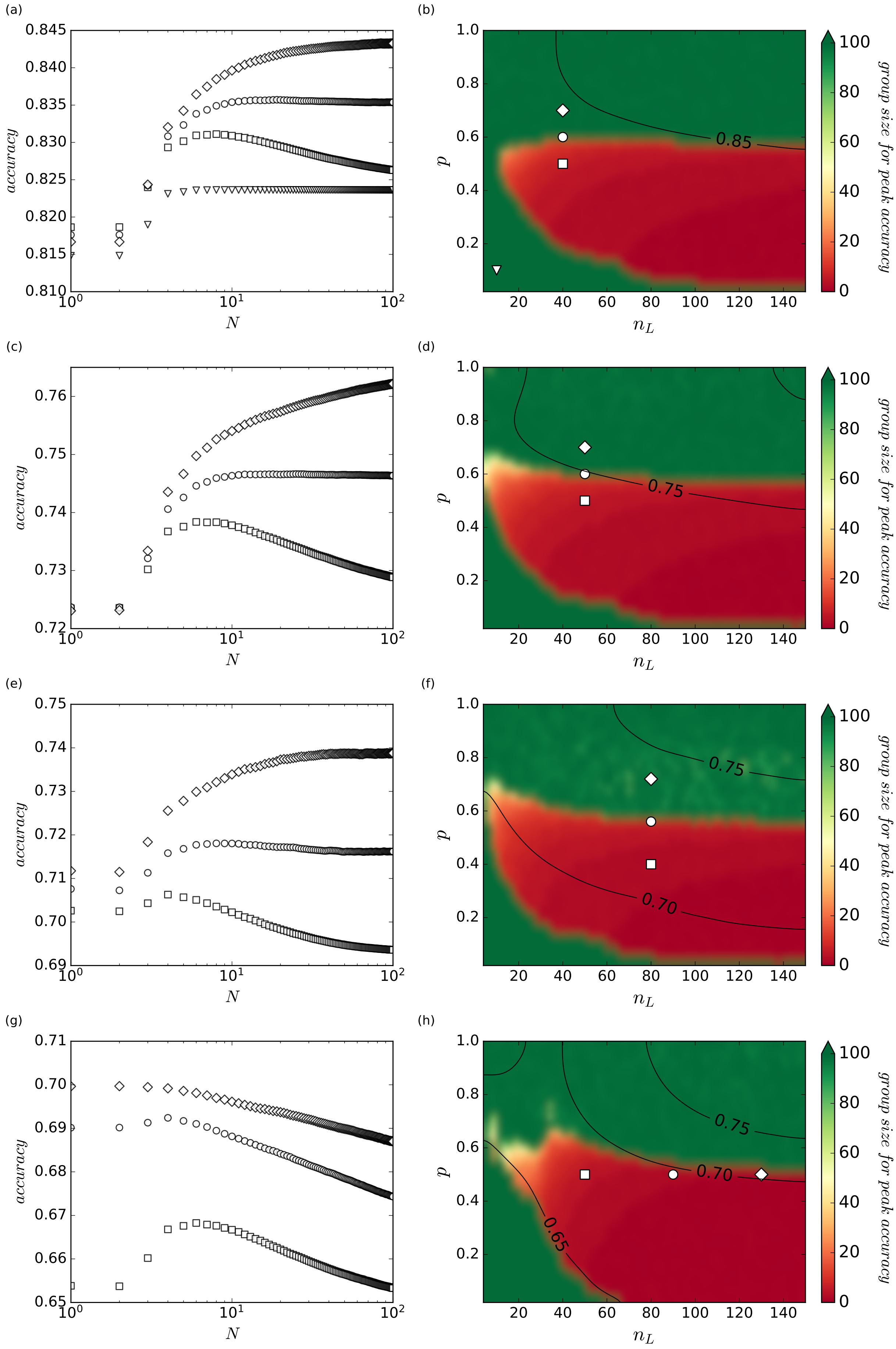

Figure S1: Collective accuracy varies depending on group size (N) and the wisdom of crowds' presence. Results for SVC models based on four datasets: (a, b) Airline Passengers, (c, d) Bank Marketing, (e, f) Car Evaluation, and (g, h) Adult Census Income. ($n_H = 50$, outcomes averaged over 200 runs of simulation). The figure details are identical to that of Fig. 5 in the main text.

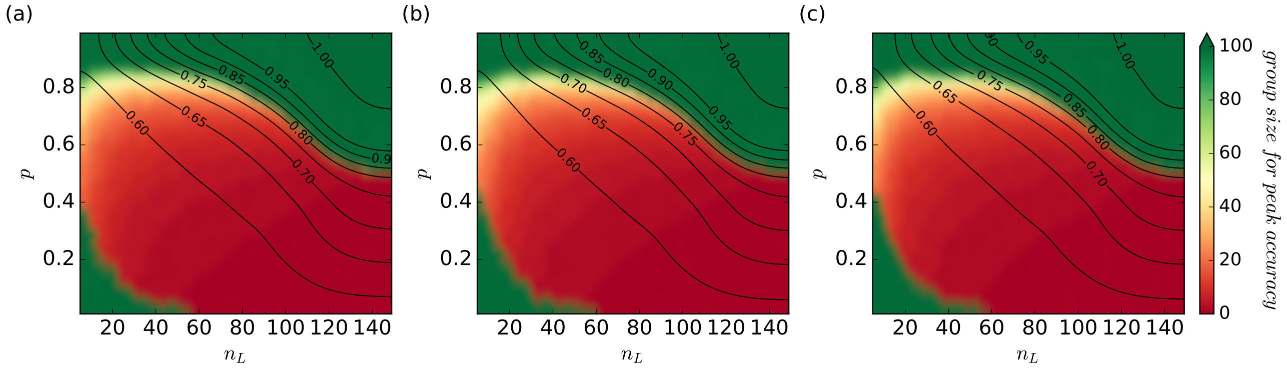

Figure S2: Analogous heatmaps to the one shown in Fig. 5b in the main part of the paper, generated for different partitions of the Circles Clusters dataset: (a) 3:7, (b) 5:5, (c) 7:3.

Fig. S2 illustrates how the division of the Circles Clusters dataset into training and test subsets affects the analysis results. The panels (a), (b) and (c) correspond to 3:7, 5:5, and 7:3 splits, respectively. As shown, this division does not significantly impact the conclusions drawn from the model discussed in this study.

3. Comparison of algorithms

Below, both algorithms - original one by Kao and Couzin and the one adapted by us for classification problems - are presented in a way that highlights their similarities and differences.

Algorithm 1 Model of Kao and Couzin

```
for each o M hearings do
    with probability r_H, generate the same correct cue for all dependent judges. Otherwise the cue is incorrect.
    for each of N judges do
        with probability p, make a judge independent. Otherwise make a judge dependent.
        if a judge is independent then
            with probability r_L his/her decision is correct. Otherwise it is incorrect.
        end if
    end for
    All dependent judges follow the same decision, based on the cue.
    Perform majority voting to determine the final decision in the hearing.
end for
Compute the average accuracy over all hearings.
```

Algorithm 2 ML model

```
for each o M hearings do
    for each of N models do
        with probability p, make a model independent. Otherwise make a model dependent.
        if a model is independent then
            train the model using distinct n_L training data (different for each model).
        else
            train the model using n_H training data (used to train all dependent models).
            To reduce correlation a share of (1 − α)n_H distinct (for each model) training data can be used.
        end if
    end for
    All models predict outcomes.
    Perform majority voting to determine the final decision in the hearing.
end for
Compute the average accuracy over all hearings.
```
